# National 10-year Cohort Study of Life-threatening Invasive Group A Streptococcal Infection in Children, 2013–2023

**DOI:** 10.1097/INF.0000000000004855

**Published:** 2025-05-12

**Authors:** Victoria Holdstock, Emily Heppenstall, Donna Corrigan, Andrew Eccleston, Laura Jones, Pota Kalima, Christopher Lamb, Eisin McDonald, Catherine M. McDougall, Jillian McFadzean, Kathryn McKenzie, Dawn Penman, Louisa Pollock, Kevin J. Scott, Andrew Smith, Alastair Turner, Roisin Ure, Margrethe van Dijke, Sadia Zafreen, Colin Begg, Nic Robertson, Thomas C. Williams

**Affiliations:** From the * Paediatric Intensive Care Unit, Royal Hospital for Children, Glasgow; † Paediatric Intensive Care Unit, Royal Hospital for Children and Young People, Edinburgh; ‡ Department of Paediatrics, University Hospital Wishaw, Wishaw; § Dumfries and Galloway Royal Infirmary, Dumfries; ¶ Department of Paediatric Infectious Disease, Royal Hospital for Children and Young People; ‖ Department of Medical Microbiology, Laboratory Medicine, Royal Infirmary of Edinburgh, Edinburgh; ** Public Health Scotland, Glasgow; †† Department of Pathology, Royal Infirmary of Edinburgh, Edinburgh; ‡‡ Department of Pathology, Queen Elizabeth University Hospital; §§ School of Infection and Immunity, University of Glasgow; ¶¶ Scottish Microbiology Reference Laboratories; ‖‖ College of Medical, Veterinary and Life Sciences, Glasgow Dental School, University of Glasgow, Glasgow; *** Department of Pathology, Aberdeen Royal Infirmary, Aberdeen; ††† Institute of Genetics and Cancer, University of Edinburgh; ‡‡‡ Department of Child Life and Health, University of Edinburgh, Edinburgh, United Kingdom.

**Keywords:** invasive group a streptococcus, child deaths, pediatric intensive care, vaccination

## Abstract

**Background:**

Invasive group A streptococcal disease (iGAS) is an important cause of pediatric morbidity and mortality. We aimed to describe severe, life-threatening, iGAS cases to inform critical care services planning and identify potential opportunities for early intervention to prevent progression to death.

**Methods:**

Retrospective, multicenter, national cohort study in Scotland investigating critically unwell iGAS cases ≤15 years old from October 01, 2013 to September 30, 2023. We included children and young people (CYP) who required advanced intensive care or died with iGAS as the primary cause of death. Information collected included demographics, *Streptococcus pyogenes emm* types, viral coinfections and clinical outcomes.

**Results:**

Eighty-two cases of severe, life-threatening iGAS were identified, with 20 resulting in death. The annual iGAS pediatric intensive care unit (PICU) admission rate was 0.69/100,000 CYP, with a mean annual mortality rate of 0.22/100,000. iGAS PICU admissions dropped during 2020–2021, returned to baseline in 2021–2022, and then increased sharply in 2022–2023 without an increase in death rates. Across the cohort, the predominant *emm* type was type 1. In 9.8% of cases, GAS was identified using a nonculture molecular method (specific polymerase chain reaction or 16S rRNA sequencing). Prior primary or secondary care contact was sought by 9/20 (45%) of CYP who died; there was no significant association between time-to-care to PICU and illness severity or risk of death. Viral coinfections were common and associated with higher severity scores.

**Conclusion:**

We demonstrate a significant annual burden of severe, life-threatening iGAS at the national level. High rates of viral coinfections and care-seeking before PICU admission or death, suggest potential opportunities for intervention.

Invasive GAS disease (iGAS), defined as infection of a normally sterile site such as blood or cerebrospinal fluid (CSF),^1^ remains an important cause of pediatric morbidity and mortality worldwide, although detailed epidemiological data remain scarce. The impact of pediatric iGAS was highlighted during a recent surge in the 2022–2023 season, following several years of lower GAS activity during and immediately after the SARS-CoV-2 pandemic.^2–6^ This increase in cases could be related to waning herd immunity,^7^ circulation of new lineages (particularly the *emm1*_*UK*_ subtype^8^), or an increase in GAS colonization coinciding with seasonal spikes in respiratory virus infections.^4,7,9^ Children and young people (CYP) seemed particularly at risk, with high numbers presenting to secondary care^3^ and high rates of empyema.^4^

Here, we aimed to describe the changing epidemiology and clinical presentation of severe, life-threatening pediatric iGAS in a high-income setting while contextualizing the 2022–2023 case surge. We focused on the most severe cases: those who died or who would very likely have died without critical care intervention, to use a robust case definition that would be consistent across a 10-year retrospective cohort. Understanding the factors underlying this surge in these cases will help inform critical care planning and identify opportunities for early intervention to prevent progression to death. By establishing baseline rates of severe, life-threatening iGAS, together with information on *emm* types, we also aimed to inform the development and eventual deployment of GAS vaccination, which is not yet available despite decades of research effort.^10^

Pediatric research within Scotland offers advantages of a known children-at-risk denominator and comprehensive data capture from national electronic healthcare records.^4,11,12^ In Scotland, iGAS is a notifiable organism,^13^ meaning that laboratories are statutorily obliged to send clinical iGAS isolates to the Scottish Bacterial Respiratory Infections Service (BRIS)^14^ for further characterization. In addition, this study leveraged the ability to capture detailed clinical and demographic information for all severe cases due to Scotland being served by only 2 pediatric intensive care units (PICUs) and having a single national reporting system for deaths.

## METHODS

We provide a summary of the methods here, with further details in Methods, Supplemental Digital Content 1, https://links.lww.com/INF/G223.

### Study Design and Participants

We conducted a retrospective, multicenter, national cohort study investigating critically unwell pediatric iGAS cases ≤15 years old over a 10-year period, with seasons running October 1 to September 30 in the subsequent year, starting October 1, 2013. Patients were defined as CYP requiring level 3 (advanced) intensive care, or who died with iGAS as the main cause of death; these were patients who either died or who would very likely have died without critical care intervention.

Patients were identified by matching PICU admissions with Scottish BRIS^4^ iGAS data. To capture possible missed cases, we undertook a comprehensive additional case capture process, as described in Methods, Supplemental Digital Content 1, https://links.lww.com/INF/G223. Patient demographics, clinical presentation and treatment details were collected from electronic clinical records (ECRs) or by liaison with clinicians who cared for the patient.^15,16^ Socioeconomic status was identified by assigning a Scottish Index of Multiple Deprivation (SIMD) quintile based on home postcode,^15^ where 1 represents the most deprived quintile and 5 the least deprived. We also calculated a rurality index for each patient and their estimated transport time to pediatric critical care (see Figure, Supplemental Digital Content 2, https://links.lww.com/INF/G224). The pediatric population at risk was calculated by the number of CYP within each age category from the National Records of Scotland^16^ midyear population. SIMD quintiles were established using the National Records of Scotland data^17,18^ (see Table, Supplemental Digital Content 3, https://links.lww.com/INF/G225).

### Outcomes

Disease phenotype was categorized into 3 groups: respiratory; skin, soft tissue or bone; and sepsis, multifactorial or other. Infection sources, bacterial emm types, viral real-time reverse-transcriptase polymerase chain reaction (rRT-PCR) testing results, and attribution of varicella co-infection were determined from electronic clinical records and BRIS.

Illness severity at presentation to the PICU was determined by the Pediatric Index of Mortality-3 Recalibrated (PIM3R) score.^19^ Deaths were judged potentially due to GAS if there was a positive GAS result in the 28 days before death. A review of national postmortem records with Group Streptococcus/*S. pyogenes* listed in death certificates was conducted at Scotland’s three pediatric postmortem sites, and deaths were judged to be primarily due to GAS infection identified. To validate this dataset, Public Health Scotland provided a count by year of all iGAS deaths reported by National Health Service Board Health Protection Teams using the iGAS enhanced surveillance questionnaire.

### Statistical Analysis

Statistical tests as indicated in text, Figure and Table legends were performed using Python 3.10.12 using the libraries NumPy (version 1.26.4), Seaborn (0.13.2) and Pandas (2.2.2). Methods, Supplemental Digital Content 1, https://links.lww.com/INF/G223 contains further details on statistical analyses for the relevant outcomes.

### Ethical Permissions

Ethical and data access permissions were obtained from the relevant institutions (see Methods, Supplemental Digital Content 1, https://links.lww.com/INF/G223, for details).

## RESULTS

### Summary of Cases

In a 10-year period, 63 CYP ≤15 years old required level 3 intensive care due to iGAS (see Figure, Supplemental Digital Content 4, https://links.lww.com/INF/G226). All required invasive mechanical ventilation to meet our case definition, and 41 (65%) required vasoactive support. Four (6%) required renal replacement therapy. These cases represented 0.39% of total PICU patient numbers and occupied 1475 (1.33%) of the approximately 111,195 bed days delivered in Scotland during the study period.

**FIGURE 1.**
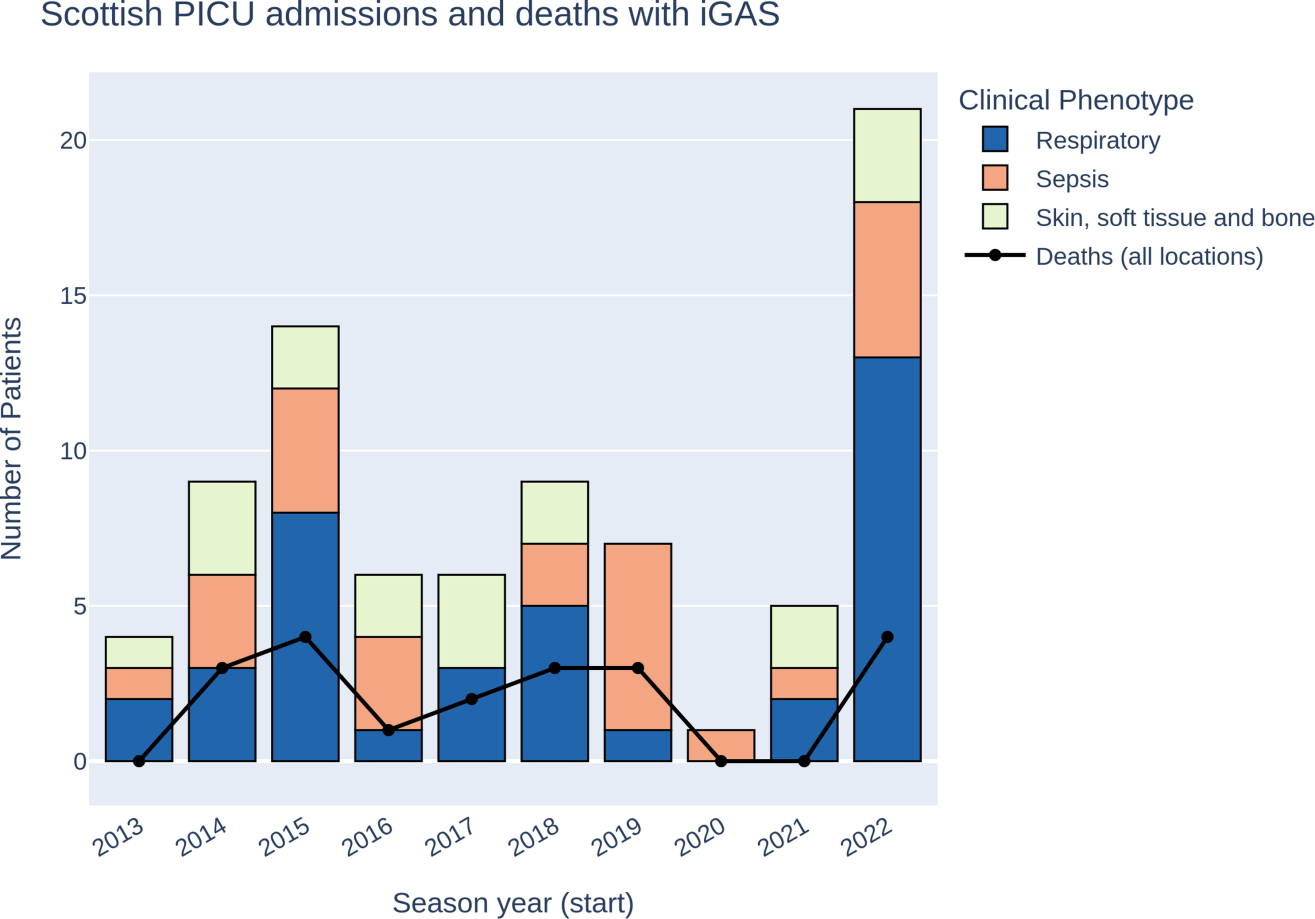
Admissions to PICU in Scotland, and deaths, due to severe iGAS infection from October 1, 2013, to September 30, 2023. Deaths are shown in black, with PICU admissions for level 3 intensive care in bars, colored by presentation phenotype (“Respiratory,” “Sepsis” or “Skin, soft tissue and bone”). iGAS indicates Invasive group A streptococcal disease; PICU, pediatric intensive care units.

One patient admitted to PICU did not survive to hospital discharge. A further 19 patients with GAS infection died before PICU admission, either in the prehospital setting, emergency department or a hospital without the 2 PICU centers. For the entire cohort, mortality was therefore 24% (20/82). Using National Health Service Board Health Protection Team reports, Public Health Scotland identified 15 deaths over the same period, a 25% underestimate of the number of deaths identified by this study. The 2022–2023 season had a higher number of cases than previous years, although the number of deaths was the same as in the 2015–2016 season (Fig. 1).

### 10-year National Trends

Throughout the study period, mean annual admission rate to PICU with iGAS was 0.69/100,000 CYP, with a mean annual mortality of 0.22/100,000 (see Table, Supplemental Digital Content 5, https://links.lww.com/INF/G227). Rates of death and PICU admission from iGAS dropped markedly during the 2020–2021 season: during the 2013–2019 seasons, there was a mean of 5.6 PICU admissions and 2.3 deaths per season, whereas, in 2020/21, there was only 1 PICU admission and no deaths (Fig. 1, see Table, Supplemental Digital Content 5, https://links.lww.com/INF/G227).

Severe iGAS rates increased sharply in the 2022–2023 season, with 17 PICU admissions and 4 deaths, a rate of 1.91 and 0.45 events per 100,000, respectively, compared with the population-corrected prepandemic (2013–2019) risk of admission of 0.61/100,000 per year, and risk of death of 0.24/100,000 per year. Admissions were significantly increased in 2022–2023 compared with the 2013–2019 seasons (*P* < 0.005 using −^2^ test), but death rates were within the expected range (*P* = 0.47). Population-adjusted death rate in the 2022–2023 season was like that in 2015–2016 (0.45/100,000 in 2022–2023 compared to 0.44/100,000 in 2015–2016).

Comparing the 40 patients in the pre-2020 seasons with the 17 admitted in the 2022–2023 season showed no significant difference in length of PICU stay, duration of ventilation, duration of vasoactive support or illness severity score (PIM3 score; see Table, Supplemental Digital Content 6, https://links.lww.com/INF/G228). There were no deaths of patients who survived to arrival at PICU in the 2022–2023 season. However, length of stay was longer than the median length of stay for all infectious diagnoses for both PICUs in 2022–2023: for the “Infection” category, this was 2.1 days in Edinburgh and 3.8 days in Glasgow,^20^ compared with a median of 6 days for iGAS admissions across both sites.

### Demographics

Comparing demographics of fatal and nonfatal severe iGAS cases (Table 1), those who survived were nonsignificantly older than those who died, with median age of 40.5 months vs. 29.5 months. There was a nonsignificant higher proportion of male patients in those who died. Comorbidity rates were similar between groups (15.0% of deaths, 17.8% of survivors; *P* = 0.78). Where data was available, there was a nonsignificantly higher proportion of non-White patients in those who died (5/20, 25%) than survived (8/62, 12.9%; *P* = 0.35). SIMD quintile distribution between survivors and nonsurvivors was also similar (see Table, Supplemental Digital Content 7, https://links.lww.com/INF/G229; *P* = 0.86 using *t* test) and there was no difference in PIM3 scores between White and non-White patients (see Figure, Supplemental Digital Content 8, https://links.lww.com/INF/G230).

**TABLE 1.** Demographic and Clinical Characteristics of the Cohort

Characteristic	PICU Survivors (n = 62)	CYP Who Died (n=20)	*P* value*
Age at admission			0.06
Median–months (IQR)	40.5 (21–67)	29.5 (11–44)	
Sex–no/total no (%)			0.25
Male	28 (45.2)	12 (60)	
Female	40 (54.8)	8 (40)	
Ethnicity–no/total no (%)			0.35
White	54 (87.1)	15 (75)	
Non-White	8 (12.9)	5 (25)	
SIMD–quintile group/total no. (%)			0.86
SIMD quintile 1-2 (most deprived)	33 (53.3)	11(55)	
SIMD quintile 3-5 (least deprived)	29 (46.7)	9 (45)	
Significant comorbidity–no/total no. (%)			0.78
Yes	11 (17.7)	3 (15)	
No	51 (82)	17 (85)	
Treatments			
Invasive mechanical ventilation	62 (100)	NA	
Vasoactive support	41 (65)	NA	
Renal replacement therapy	4 (6)	NA	

* *P* values calculated using Mann-Whitney *U* test.

IQR indicates inter-quartile range; SIMD, Scottish index of multiple deprivation; NA, not applicable.

Comparing our cohort’s (both fatal and nonfatal cases) demographics to overall Scottish demographics, a higher-than-expected proportion of included patients were in the most deprived 2 deprivation index (SIMD) quintiles: 53.8% compared with the expected 41.6% based on population data from the cohort mid-point in 2018 (see Table, Supplemental Digital Content 3, https://links.lww.com/INF/G225). This skew towards more deprived patients being overrepresented was statistically significant when comparing the overall SIMD quintiles distribution between the cohort and the pediatric population of Scotland as a whole (*P* = 0.03 using a χ^2^ test).

Regarding ethnicity, 5.8% of the Scottish pediatric population was non-White in the 2011 census, whereas 13/82 (15.9%) of the cohort was non-White, therefore overrepresented (*P* = 0.0003). However, the proportion of non-White CYP in the Scottish pediatric population increased to 11.8% in the 2022 census; repeating the analysis using the 2022 data resulted in a nonsignificant difference (*P* = 0.34). Therefore, although non-White CYP appear to be overrepresented, low case numbers and a changing Scottish ethnic make-up over time limit our ability to draw conclusions about the significance of this finding.

### Role of Molecular Diagnostics

In total, 8 cases (9.8%) had iGAS confirmed by a nonculture method. The first molecular iGAS diagnosis was made in 2015, with a positive iGAS RT-PCR on pleural fluid. Since then, 2 cases were identified by blood RT-PCR, 1 on pleural fluid RT-PCR and 5 by 16S rRNA sequencing (see Table, Supplemental Digital Content 9, https://links.lww.com/INF/G231 and Figure, Supplemental Digital Content 10, https://links.lww.com/INF/G232). In the 2022–2023 season, 2/17 (12%) iGAS diagnoses in PICU admissions were made using molecular diagnostics, implying that cases may have been missed earlier in the cohort period when these were not so readily available.

### *emm* types

Of the 21 cases of severe pediatric iGAS in 2022–2023, 15 (71%) were *emm* type 1, compared with an average of 38.8% (range 22.2–50%) in the pre-2020 years (see Figure, Supplemental Digital Content 11, https://links.lww.com/INF/G233). The second most common *emm* type in the 2022–2023 season was 12 (10% of severe cases), consistent with the prepandemic trend where, on average, 10% of cases were *emm* type 12 (range 0–33%). The increased proportion of *emm* type 1 versus other isolates among the typed samples in 2022–2023 was significant when compared with the pre-2020 trend (*P* = 0.006 using χ^2^ test).

Across the study period, *emm* type 1 was more commonly associated with respiratory (56.8% of cases) than other phenotypes (sepsis, 24.3%; skin, soft tissue and bone, 18.9%; *P* = 0.046 using χ^2^ test).

### Viral coinfections

**FIGURE 2.**
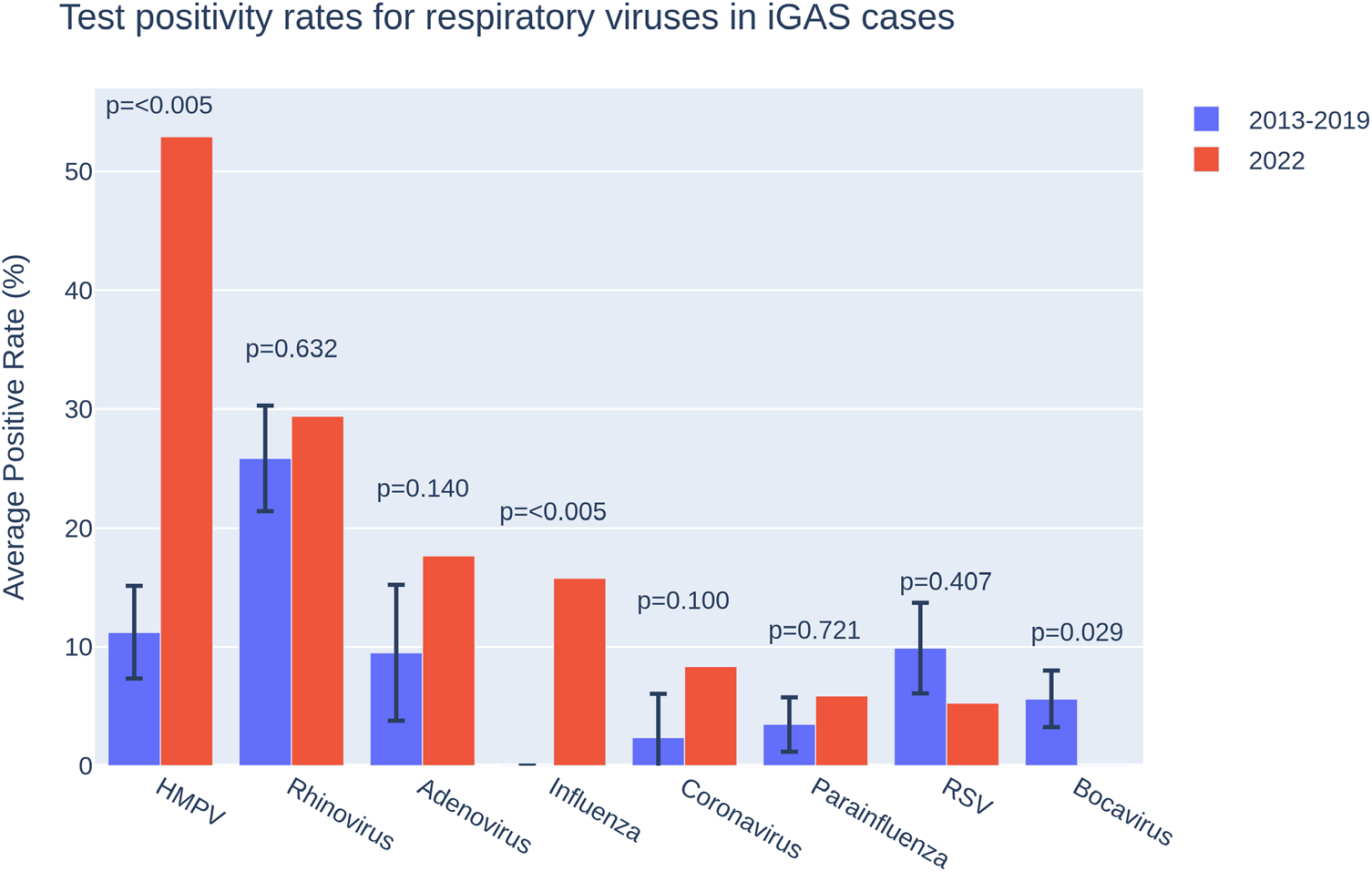
Virus positivity on rRT-PCR testing. Error bars are standard error, with *P* values derived from Fisher exact tests on odds ratios. iGAS indicates Invasive group A streptococcal disease; HMPV, human metapneumovirus; rRT-PCR, real-time reverse-transcriptase polymerase chain reaction.

The most commonly detected viruses were rhinovirus (18 positive of 63 tests, 28.6%) and human metapneumovirus (HMPV; 16 positive of 61 tests, 26.2%; see Figure, Supplemental Digital Content 12, https://links.lww.com/INF/G234). Test positivity rates varied over time (Fig. 2). Most notably, test positivity rates for HMPV increased markedly in the 2021–2022 (33.3%) and 2022/23 (52.9%) seasons, compared with both a pre-2020 average (11.2%) and a previous peak (28.6% in 2019). The increased HMPV positivity rate in 2022/23 was raised compared with the 2013–2019 seasons (*P* = 0.007 using χ^2^ test). This result is unlikely due to increased testing: in 2022, 17/21 patients had HPMV testing (81.0%) compared with 41/55 patients in prepandemic years (74.6%), a nonsignificant difference (*P* = 0.78 using χ^2^ test). SARS-CoV-2 rRT-PCR testing was available in PICUs from March 2020 onwards, but no patients eligible for testing (27 cases) were positive. This low rate of SARS-CoV-2 co-infection is in keeping with UK-wide data on empyemas from the 2022–2023 season.^21^

Overall, patients with iGAS and respiratory virus coinfection were more unwell on arrival at PICU. Of 62 patients with a PICU admission PIM3 score, the mean score was 0.05 for 32 patients with ≥1 proven respiratory viruses, compared with 0.03 for 30 with no confirmed viruses (*P* = 0.016 for Mann-Whitney *U* test; see Figure, Supplemental Digital Content 13, https://links.lww.com/INF/G235). Although the mean PIM3 score varied for different viruses, this was not significant (*P* = 0.37 using an analysis of variance test; see Figure, Supplemental Digital Content 14, https://links.lww.com/INF/G236).

Varicella zoster virus infection is a known precipitant of iGAS,^22,23^ but varicella zoster virus rates in our cohort were not increased in 2022 compared with the prepandemic trend. Around 19.1% of cases in the 2022–2023 season were positive either by viral PCR testing or clinical impression, compared with 25.5% previously (p=0.76 using χ^2^ test).

### Time-To-Care and Outcomes

Scotland has a geographically diverse population, with many CYPs living far from the 2 PICU centers (see Figure, Supplemental Digital Content 2, https://links.lww.com/INF/G224). We tested whether patients with iGAS with a potentially longer journey to intensive care were more likely to die or have higher illness severity scores. Of the 79 cases where data was available across the whole cohort, 51 (64.6%) had a time-to-care of ≤1 hour, while 14 (17.7%) had a time-to-care of >2 hours. Overall, 73.7% of patients who died had a home address >30 minutes away from the PICU, compared with 66.7% of patients who survived (*P* = 0.93 using χ^2^ test). There was a trend for admission PIM3 scores to increase as time-to-care increased, but this was also not significant (Spearman’s correlation of 0.085).

### Prior Clinical Review

For patients who died, 45% had sought care before their final attendance (15% in primary care and 30% in secondary care). For those who were admitted to PICU and survived, 13.7% had sought care in a secondary care facility before the attendance resulting in PICU admission. The comparison of the 2 groups is limited by the fact that we did not have information on primary care attendances for those who were admitted to PICU and survived.

## DISCUSSION

### Changing Epidemiology of iGAS, Social Determinants of Health, and Role of Viral Coinfections

Our data provide incidence rates of severe iGAS disease in our national population using all known cases over a 10-year period, along with comprehensive information on ethnicity and socioeconomic status. The GAS surge during 2022–2023 had a higher number of severe iGAS cases than previous years but the same number of deaths. Over time, severe disease increasingly presented with a respiratory phenotype, with *emm* type 1 predominating. As previously reported,^2–6^ the majority of the 2022–2023 cases were caused by an increase in *emm* type 1 isolates. Our data also highlights the importance of access to molecular diagnostics, without which about 10% of cases of severe iGAS would not have been identified.

This study adds to other work illustrating that low socioeconomic status is a risk factor for pediatric intensive care admission,^24^ and that non-White ethnicity may be a risk factor for iGAS disease^25^ and death. This information could be used to target public health intervention strategies and design primary prevention interventions.

CYP with severe iGAS who were admitted to PICU or died often had presentations to healthcare professionals in the period immediately before their index illness. This suggests that we captured these case presentations at different stages of the chain of survival^26^: as the excellent prognosis of PICU patients demonstrates, each death can be seen as a missed upstream opportunity for earlier identification of critical illness. There is a need for further research to identify those CYPs at the highest risk of deterioration and to identify potential opportunities for early intervention. We did not find an association between distance to PICU and severity of illness on arrival or death, providing reassurance that Scotland’s pediatric critical care infrastructure is not systemically disadvantaging patients living in more rural areas.

Viral co-infection was common and suggests a potential role for viral immunization in preventing severe iGAS disease. Varicella zoster virus is now part of the UK immunization schedule,^27^ and live respiratory syncytial virus vaccines for older infants and toddlers are in development.^28^ HMPV vaccines were in development but following safety concerns in a recent trial may take longer to enter clinical practice.^29^ While viral coinfection may be implicated in severe iGAS, not all cases demonstrated viral coinfection, and this area of microbial ecology requires further exploration. Of note, there were no SARS-CoV-2 coinfections in this cohort.

Once a CYP with severe iGAS was admitted to the PICU, they had greater critical care morbidity than the baseline PICU population, as evidenced by the level and length of organ support required, but a very low risk of death. This information is useful for clinicians when counseling the carers of CYP admitted to PICU and provides a potential metric to calculate the health-economic benefits of any future vaccination campaign against GAS. However, our findings contrast with those from a study from Canada from the 2022 to 2023 season, which showed a much higher mortality rate for pediatric patients with iGAS admitted to the PICU (20%).^30^ Understanding country-specific factors in care-seeking and access to care will be key in designing interventions to reduce the impact of this condition.

### Strengths and Limitations of the Study

We present a large national dataset including all severe, life-threatening, iGAS cases over a 10-year period using a thorough and multi-pronged approach to case identification, a known population denominator, and including complete information on clinical course, ethnicity and socioeconomic status. Due to our stringent inclusion criteria, our results are likely to underestimate the incidence of severe disease; for example, our cohort does not include cases of empyema not requiring intensive care admission, or sepsis not requiring ventilatory support. Our results, therefore, do not provide an overview of the burden of iGAS in CYP in Scotland but rather only the burden of the most severe cases.

### Considerations for Future Work

There is a need for longitudinal follow-up of survivors of severe critical iGAS illness to assess long-term effects on respiratory, cardiovascular and neurocognitive functioning. Host genomic studies may help to identify CYP at particular risk of further deterioration, and bacterial genomics may identify factors associated with more severe disease. More research is also needed to understand the association between socioeconomic status and critical illness. Prospective work in this area should include data on patient ethnicity.

Our study highlights the limitations of existing public health surveillance systems (only 75% of deaths in our study were identified by current surveillance mechanisms) and the need for robust data sharing going forward, including information on deaths. Such datasets would inform policy planning and reassure parents/carers of those who have died that lessons are being learned.

In conclusion, our study identifies potential areas for intervention to reduce the burden of severe iGAS, which remains a significant cause of morbidity and mortality worldwide.

## ACKNOWLEDGMENTS

*The authors thank Nicola Kirby for her assistance in querying the PICU admissions database in Glasgow. We acknowledge the work of Kate Templeton and her team for developing the GAS RT-PCR testing used in NHS Lothian. We acknowledge the work of staff at the Scottish Microbiology Reference Laboratory (Glasgow) on emm typing of S. pyogenes isolates and the development of a GAS RT-PCR testing protocol. The 16S rRNA gene and S. pyogenes rRT-PCR processing and reporting of specimens from some patients reported in this study was conducted by the Microbiology Laboratory at Great Ormond Street Hospital (Julianne Brown, Laura Atkinson, and Timothy Best), and for others by the UKHSA Colindale Bacterial Reference Service. We thank Ryan McHenry, QEUH Emergency Department for R expertise and assistance with Statistics Scotland API search strategy and Jon McCormack, RHCYP Edinburgh for their time in creating the Paediatric Critical Care map. We thank Alan Coventry Information Analyst, David Carr, Principal Information Analyst & Salomi Barkat, Senior Information Analyst, Public Health Scotland: Elizabeth Fraser, Poverty and Deprivation Statistics, Equality and Social Justice Analysis, Scottish Government Communities Analysis Division, Edinburgh, Dr Hannah K Mitchell, Institute of Child Health, University College London (all for advice on deprivation metrics). We thank Clare Webster (Ninewells Hospital Dundee) for assistance in data collection.*
